# Clival chordoma presenting with isolated unilateral cranial nerve XII palsy: A case report

**DOI:** 10.1016/j.ijscr.2024.109393

**Published:** 2024-02-15

**Authors:** Nahar Ismaiel, Leen Jaloud, Tima Barhoum, Rami Issa, Zuheir Alshehabi

**Affiliations:** a Cancer Research Center, Tishreen University, Latakia, Syria; b Department of Pathology, Tishreen University Hospital, Latakia, Syria; c Faculty of Medicine, Tishreen University, Latakia, Syria

**Keywords:** Chordoma, Cranial nerve XII palsy, Dysarthria, Surgical resection, Case report

## Abstract

**Introduction:**

Chordomas are rare malignant neoplasms that can originate anywhere along the cerebrospinal axis. However, they are most commonly found in the spine, cranium, and sacrococcygeal region. Chordomas can manifest differently depending on their location and most symptoms are a result of local invasion. We present a rare case of intracranial clival chordoma that manifested as isolated unilateral cranial nerve XII (CN XII) palsy.

**Presentation:**

A 53-year-old male presented to the neurosurgical clinic with headaches, dysarthria, and pharyngeal pain. Neurological examination showed left-sided atrophy of the tongue. MRI scan showed an infiltrative lesion in the clivus which was hypointense on T1 and hyperintense on T2. The lesion was treated surgically however full resection was not achieved. Pathological examination and subsequent immunohistochemical staining confirmed the diagnosis of chordoma.

**Discussion:**

To our knowledge, there have been only two reported cases of clival chordoma that presented with isolated CN XII palsy which manifested clinically as dysarthria and unilateral atrophy of the tongue. This makes our case the third reported case of clival chordoma that presented with isolated CN XII palsy.

**Conclusion:**

We report a rare case of clival chordoma that presented with isolated left CN XII palsy. Physicians should consider clival chordomas in their differential diagnoses when faced with isolated unilateral CN XII palsy. Surgical resection combined with adjuvant radiotherapy remains the preferred treatment protocol.

## Highlights

• Chordomas are rare neoplasms that can be found anywhere along the neuraxis.
• Symptoms of cranial chordomas are varied and they are mostly attributed to local invasion.
• There have been only two reported cases of clival chordoma that presented with isolated unilateral CN XII palsy.
• Various imaging modalities can be used to study these tumors, such as plain radiographs, CT scans, and MRI scans.
• Surgical resection followed by adjuvant radiotherapy is the preferred treatment protocol.

## Abbreviations

CN XII: Cranial Nerve XII
CT: Computerized Tomography
MRI: Magnetic Resonance Imaging
CK: Cytokeratin
EMA: Epithelial Membrane Antigen

## 1 Introduction

Chordomas are rare, slow-growing, locally invasive malignant neoplasms that are usually derived from primitive notochord remnants (1,2). These lesions can be found anywhere along the neuraxis and they usually have a small male predominance (1).

Chordomas are usually located in the spine followed by the cranium, the sacrococcygeal region is the least common location (1,3).

Symptoms of chordoma occur mostly due to local invasion and are varied depending on the location of the tumor (2,4). Only two cases reported the presence of isolated unilateral cranial nerve XII (CN XII) palsy as a result of clival chordoma (5,6). The main imaging modalities used for diagnosing this condition are computed tomography (CT) scan and magnetic resonance imaging (MRI) scans (5).

Herein we report, to our knowledge, the third known case of clival chordoma that presents with isolated CN XII palsy. This case report has been reported in line with the SCARE criteria (7).

## 2 Presentation

A 53-year-old male presented to the neurosurgical clinic at our hospital with severe headaches, dysarthria, and pharyngeal pain. Upon further interrogation, it was found that the pharyngeal pain started 8 months ago and the patient also mentioned that he had lost weight over the past few months. The patient had no past medial or surgical history. Neurological exam showed left-sided atrophy of the tongue and dysarthria which was consistent with left CN XII palsy and the exam was otherwise normal. Laboratory tests were ordered and all were within the normal range.

**Fig. 1:**
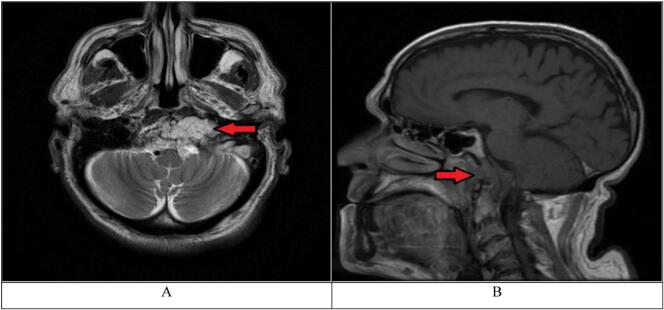
A. T2-weighted MRI, axial view. The arrow shows hyperintense Clival lesion located 3 mm from the anterior end of the clivus, the mass can be seen compressing the left internal carotid artery. (pre-operative MRI). B. T1-weighted MRI, sagittal view. The arrow shows hypointense mass causing severe destruction of the clival cortex, the mass can be seen compressing the anterior aspect of the medulla. (pre-operative MRI).

The patient was admitted to the neurosurgical department for monitoring and an MRI scan was ordered (Fig. 1). The scan showed an infiltrative lesion in the clival bone marrow which was hypointense on T1 and hyperintense on T2 weighted images. This lesion was located 3 mm from the anterior end of the clivus extending 35 mm laterally to the left accompanied by significant damage to the clival cortex on both the anterior and posterior aspects. The scan also shows the lesion infiltrating the dura of the brainstem compressing the anterior left aspect of the medulla and pushing the left internal carotid artery medially. After the administration of gadolinium, the scan showed mild enhancement within and around the clival region.

The medical team decided that surgery was the best method of treatment for this patient and the main goal was to achieve sufficient debulking of the tumor and to try resecting it fully.

The surgery was done using an endoscopic endonasal approach. Sufficient dissection was performed until the clivus was reached and the tumor capsule was visible. The capsule was subsequently opened and the tumor was debulked and resected successfully in a piecemeal fashion along with parts of the clivus where the tumor originated. However, total resection was not achieved.

Post-operatively, the patient improved and his symptoms subsided.

**Fig. 2:**
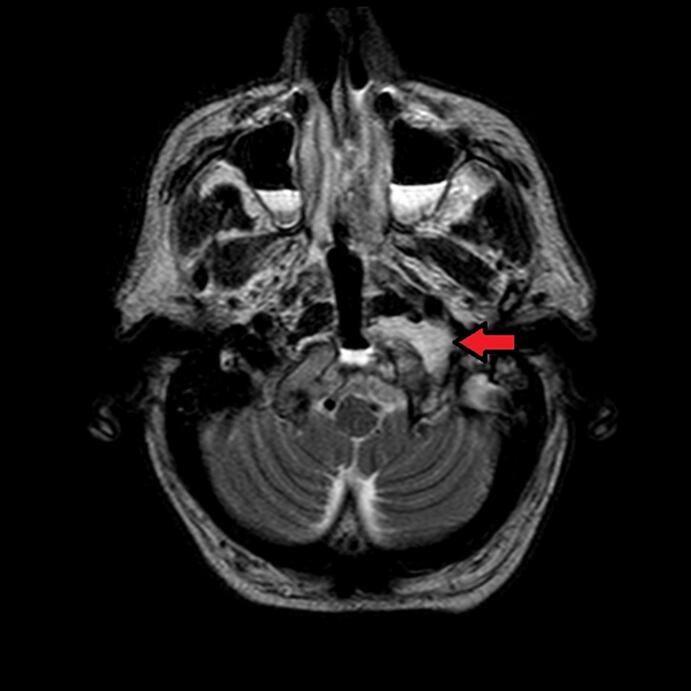
T2-weighted MRI, axial view. Signs of surgical intervention on the clivus are noted. The arrow shows residual tumor. (post-operative MRI).

Post-operative MRI (Fig. 2) showed signs of surgical intervention on the clivus in addition to residual tumor.

Gross examination revealed several fragments of tan soft tissue measuring in aggregate.

2 × 1.5 × 0.5 cm.

**Fig. 3:**
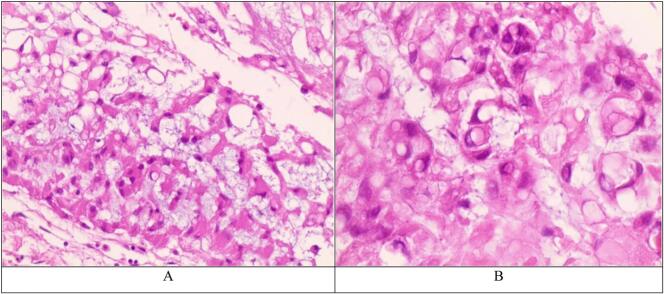
A. Chordoma (H&E 100×). Prominent myxoid background containing small columns or clusters of bubbly physalipharous cells. B. Chordoma (H&E 400×). Occasional cells with irregular or hyperchromatic nuclei.

Pathological examination showed a prominent myxoid background containing small clusters, columns, and individual neoplastic cells that have relatively well-defined borders and significantly vacuolated or eosinophilic cytoplasm (bubbly physalipharous cells). Occasional cells with slightly irregular or hyperchromatic nuclei are noted. (Fig. 3).

**Fig. 4:**
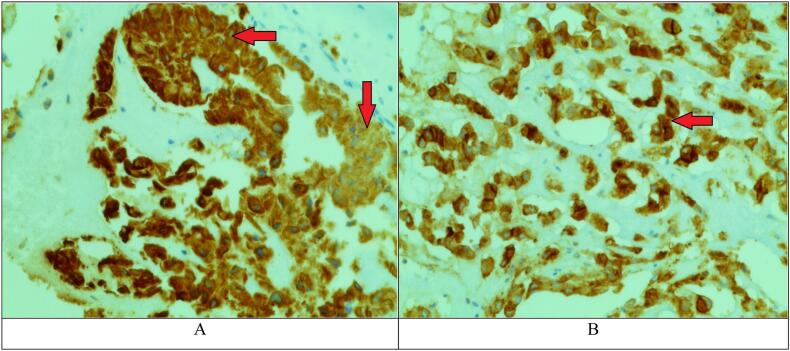
A. Chordoma, Immunohistochemical staining. Arrows show positivity for cytokeratin (CK). B. Chordoma, Immunohistochemical staining. The arrow shows positivity for epithelial membrane antigen (EMA).

Immunohistochemical staining (Fig. 4) was ordered and it showed positivity for cytokeratin (CK) and epithelial membrane antigen (EMA) confirming the diagnosis of chordoma. S100 was not ordered as it was not available in our hospital.

The patient was referred to an oncologist and was put on a radiotherapy treatment plan. His condition remains under control.

## 3 Discussion

Chordomas are rare neoplasms accounting for 1–4 % of all bone malignancies. They have an incidence rate of 0.08 per 100,000 with a small male predominance (8).

Although these lesions can be found anywhere along the neuraxis, they are most commonly located in the spine followed by the cranium, and finally the sacrococcygeal region (1,3). Intracranial chordomas can commonly arise from the clival region, the spheno-occipital synchondrosis, and the Sella Turcica (2,4).

Due to the slow growing nature of these tumors; chordomas usually remain clinically silent until the later stages of disease progression (8). And when they are expressed clinically symptoms vary depending on the location of the lesion (1,2,8).

Symptoms of cranial chordomas are mostly attributed to local invasion of the surrounding delicate structures and they may include: Headaches, neck and pharyngeal pain, Cranial neuropathies (the most common of which is cranial nerve VI palsy), diplopia, and facial numbness.

Other less common symptoms include nasal obstruction, subarachnoid hemorrhage, chronic cerebrospinal fluid rhinorrhea, and hypopituitarism if the Sella was involved (1,2,4,5,9).

To our knowledge, there have been only two reported cases of clival chordoma that presented with isolated unilateral CN XII palsy (5,6), making our case the third reported case of clival chordoma that presented with isolated unilateral CN XII palsy in the literature which was expressed clinically as dysarthria and unilateral atrophy of the tongue.

Various imaging modalities can be used to study these tumors, such as plain radiographs, CT scans, and MRI scans (1,2).

Plain radiographs show bony erosion and scattered calcifications (1). CT scans paint a more complete picture. On non-contrast CT scans, chordomas appear as well-circumscribed, hypoattenuating, heterogeneous lesions accompanied by extensive lytic bone destruction (10).

On T1-weighted MRI images, chordomas can show variable signal intensity. However, they are generally hypointense with the presence of small foci of hyperintensity mainly due to mucus or hemorrhage (10). On T2 weighted images these tumors are mainly hyperintense with additional small foci of hypointensity which can again be attributed to mucus, hemorrhage, or calcifications (10).

Finally, MRI scans are usually preferred over CT scans when studying these tumors as MRI scans can provide a better distinction of the lesion and a more accurate assessment of the surrounding vasculature (2).

Although CT and MRI scans can be helpful in diagnosing chordomas, pathology and immunohistochemistry are the golden standard for diagnosing these lesions. Pathologically chordomas can be divided into 3 subtypes; conventional, chondroid, and dedifferentiated (1). Conventional chordomas (as in our case) usually show bubbly physalipharous cells with a myxoid stroma. These lesions can sometimes exhibit cellular proliferation which appears as numerous mitotic figures, nuclear pleomorphism, and the presence of nucleoli (1). Immunohistochemical stains demonstrate reactivity for cytokeratin (CK), epithelial membrane antigen (EMA), Vimentin, and S100 (1,8). This matches our pathological findings which were discussed previously in the presentation.

Treatment for intracranial chordomas is a challenge mainly due to these lesions often being located near sensitive and delicate anatomical structures such as the brain stem, pituitary gland, optic chiasm, and other vital structures which makes accessing them a challenge for surgeons (2,5).

the main method of treatment used for intracranial chordomas is surgical resection which can be done with different approaches according to the surgeon's preference.

Common approaches include trans-sphenoidal, trans-maxillary, trans-nasal, high anterior cervical retro-pharyngeal, trans-cochlear, trans-mandibular, and transoral (1,8).

However, for clival lesions, the preferred surgical approach is the endoscopic endonasal approach due to it being minimally invasive which results in fewer complications (1).

Although surgical resection is essential in treating chordomas, usually gross total resection cannot be achieved (8). In these cases, subtotal resection (with emphasis on neurological preservation) in addition to adjuvant radiotherapy, mainly proton beam therapy, (as most chordomas are resistant to chemotherapy) is the accepted treatment protocol as it achieves satisfactory control of the disease (1,2,8).

## 4 Conclusion

Chordomas are rare, slow-growing, locally invasive malignant neoplasms that can arise anywhere along the cerebrospinal axis. Herein we presented the 3rd known case of clival chordoma manifesting as isolated unilateral CN XII palsy.

This case shows that, despite the rarity of this presentation, chordomas of the clival region can sometimes manifest as isolated cranial nerve XII palsy. As a result, physicians must consider chordomas in their differential diagnoses when presented with isolated unilateral cranial nerve XII palsy.

In these cases, surgical resection followed by adjuvant radiotherapy proved to be the best treatment protocol as it can achieve long-term control of this disease thus improving the quality of life of those who are suffering from it.

## Consent

Written informed consent was obtained from the patient for publication of this case report and accompanying images. A copy of the written consent is available for review by the Editor-in-Chief of this journal on request.

## Provenance and peer review

Not commissioned, externally peer-reviewed.

## Ethical approval

Given the nature of the article, a case report, no ethical approval was required.

## Funding

No funding was required.

## Author contributions

All authors contributed to this manuscript.

Nahar Ismaiel: Writing - original draft, reviewing, and editing.

Leen Jaloud: Writing - original draft, reviewing, and editing.

Tima Barhoum: Writing - original draft, reviewing, and editing.

Rami Issa: Writing - original draft, reviewing, and editing.

Zuheir Alshehabi: Supervision; final reviewing and editing.

## Guarantor

Prof. Zuheir Alshehabi.

## Research registration number

Not needed.

## Declaration of competing interest

The authors declare no conflict of interest.
